# Development of a new operative 3D printed template-cranioplasty-concept for performing craniotomy in the posterior cranial fossa and validation of the system using a 3D printed simulation model: a preclinical feasibility study

**DOI:** 10.1186/s41205-026-00332-y

**Published:** 2026-06-18

**Authors:** Svenja Jung, Maike Stummer, Christian König, Erdem Güresir, Dirk Winkler, Felix Arlt, Ronny Grunert

**Affiliations:** 1 Department of Neurosurgery, University of Leipzig, Leipzig, 04103 Germany; 2 Institute of Forensic Medicine, University of Leipzig, Leipzig, 04103 Germany; 3 Fraunhofer Institute for Machine Tools and Forming Technology, Zittau, 02763 Germany

**Keywords:** 3D print, 3D printed template, Neurosurgical, Cranioplasty, Fossa cranii posterior, Retrosigmoid approach, Additive manufacturing

## Abstract

**Background:**

The retrosigmoid approach is a standard surgical route used for the treatment of various tumors and vascular lesions in the cerebellopontine angle. However, postoperative reconstruction of the surgical defect could remain challenging, particularly in cases requiring intraoperative extension. For this reason, a novel concept was developed to define the surgical approach and enable precise defect coverage using a patient-specific implant. This concept was designed to be patient-specific and realized through 3D printing.

**Methods:**

The developed 3D printed template-implant concept was designed to facilitate precise craniotomy. The template is used as a stencil and a surgical marker is applied to delineate the implant contours directly on the patient’s skull. This outline subsequently serves as a guide for where to perform the craniotomy and defines its boundaries. The custom-fit implant replicates the template design and ensures complete coverage of the craniotomy defect, while allowing intraoperative adjustment if required. Preclinical testing was conducted on a 3D printed simulation model representing the posterior cranial fossa (surgical area). The novel concept was tested by nine senior and attending neurosurgeons from the University Hospital Leipzig. Bilateral craniotomies were performed using the template and subsequently closed with the implant. The fit of the implant was evaluated using CT scans. The time required for preparation, including craniotomy, as well as for post-procedure handling, including implant placement, was recorded.

**Results:**

The average gap of the new concept was 2.11 mm, which was significantly smaller than that of the current standard approach (5.52 mm) and showed lower variability. The entire procedure, including craniotomy and implant placement, took an average of 13 min and 20 s.

**Conclusion:**

The novel template-implant concept for retrosigmoid approaches improves defect coverage and reduces gap sizes. Furthermore, it demonstrates the potential of 3D printed patient-specific implants for more precise and predictable surgical procedures, although further studies are required to validate efficiency and clinical safety.

## Background

The retrosigmoid approach is a standard surgical route used for the treatment of various tumors and vascular lesions in the cerebellopontine angle [1]. Despite its widespread use and clinical efficacy, postoperative reconstruction of the surgical defect can present a challenge due to intraoperative extensions.

A key issue motivating this study is the incomplete coverage of the defect created during surgery. Accordingly, the aim was to define the retrosigmoid approach using a novel template and to achieve complete defect coverage with a 3D printed cranioplasty based on this template. The utilization of templates to facilitate surgical procedures has a well-established presence in various medical specialties. In vascular surgery, for instance, templates are employed to pre-cut stents prior to surgical interventions [2, 3]. A similar application can be observed in bone surgeries, including osteosarcoma removal [4, 5], tumor excisions [6, 7], osteotomies [8] and maxillofacial procedures [9]. More frequently, templates are utilized to precisely position rods or screws, such as in femoral surgeries [10–15]. In neurosurgery, such techniques are also applied, notably for spinal stabilization [16, 17]. However, the use of templates as cutting guides in skull surgery is not widespread. A more common application involves 3D printed templates and molds for creating precise implants based on pre-existing defects [18–24]. These defects are typically extensive and located on the cranial vault. For smaller, occipitally located cranial defects, applications have been described in which a 3D printed cranioplasty provides precise defect coverage [25]. However, the use of a guiding template has not been reported.

The objective of this work was to develop a defined retrosigmoid surgical approach and its precise coverage using 3D printing technology. The proposed concept includes a template that can be transferred to the skull bone to define the surgical access route. Subsequently, the defect will be covered using a precise, 3D printed cranioplasty. The novel concept was evaluated by neurosurgeons using a previously developed simulation phantom, representing a purely preclinical feasibility study, with further investigations on cadaveric specimens planned for future validation.

## Methods

**Fig. 1:**
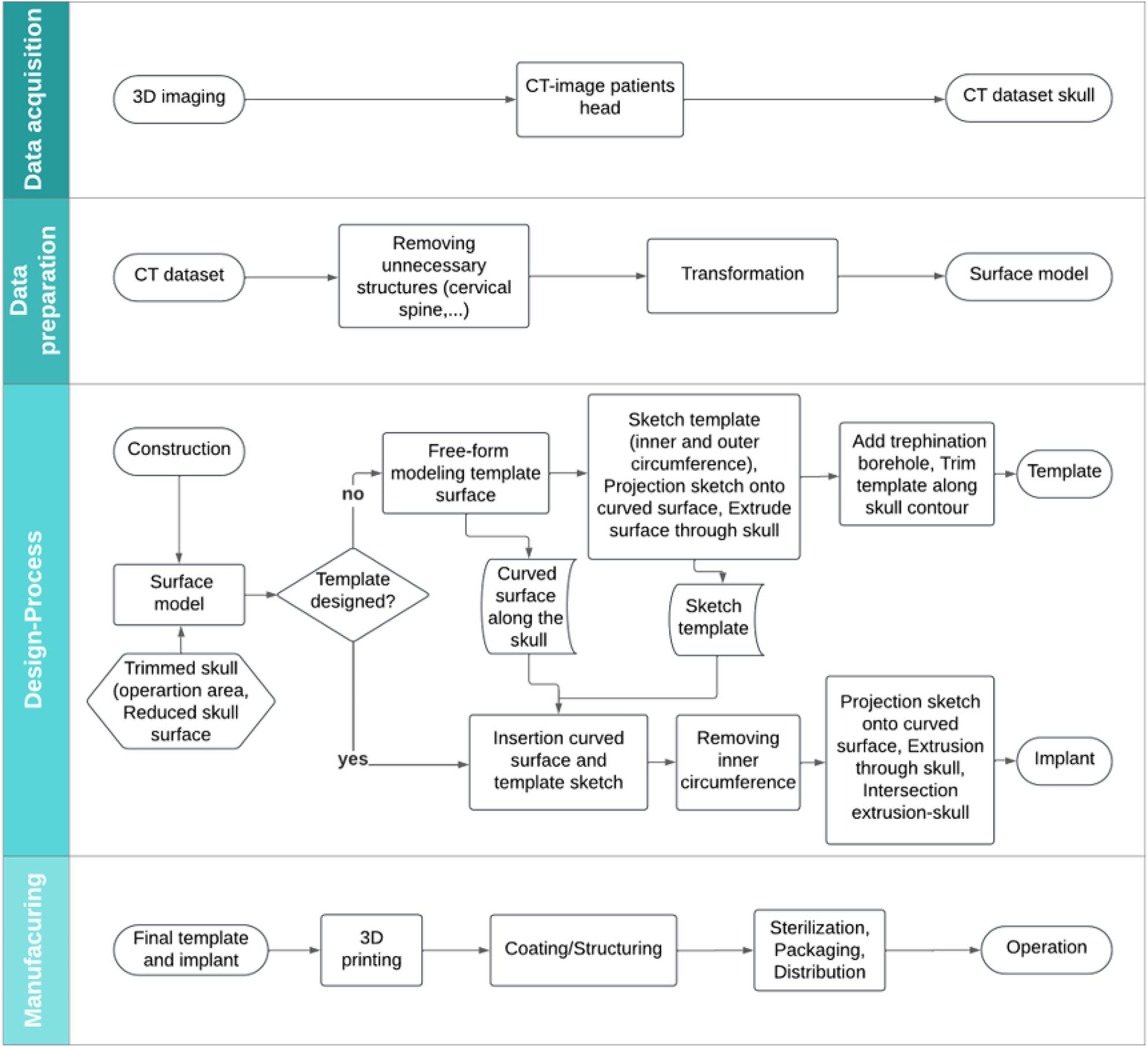
Overview of the workflow for the development of the newly designed patient-specific template-implant concept for the posterior cranial fossa

As illustrated in Fig. 1, the creation of the patient-specific template-implant-concept is a process that can be subdivided into four stages. The implantation of the implant itself is the only stage that occurs in the operating room.

The ensuing chapters elaborate on the design, the fabrication of the template and implant, and the evaluation of the novel concept in relation to the current standard of care.

### Functionality of the novel template-implant concept

The developed concept provides the surgeon with a patient-specific template for the craniotomy, which can be accurately positioned on the patient’s skull. The contours of the template are first transferred onto the skull. For the actual craniotomy, the template is removed, and the surgeon follows the marked lines. The outer contour of the template defines the maximum extent of the craniotomy and should not be exceeded, while the inner contour establishes the minimum boundary and must not be undercut.

The patient-specific implant corresponds exactly to the shape and dimensions of the template, allowing complete coverage of the resulting defect when the craniotomy is performed to its maximum extent. If the craniotomy is smaller than the outer contour of the template, the implant can be adjusted accordingly to ensure precise defect coverage. Conversely, a craniotomy exceeding the outer contours of the template cannot be fully covered by the implant.

### Clinical requirements for the novel concept

The requirements for the novel concept were developed in collaboration with an experienced neurosurgeon (F.A.). Key aspects included the current surgical procedure for a retrosigmoid approach and specific requirements for the template, implant, and material.

For the planning and positioning of the template and implant, as well as for the creation of the 3D printed simulation model, the same STL data were used. These were based on a 1 mm CT scan of a real patient, which had been previously segmented. The Hounsfield unit threshold ranged between 224 and 3071. Due to the large data size and subsequent processing steps required for the developed phantom, the model had to be reduced. Accordingly, the planning and positioning of the template and implant were also performed on the reduced model. For future testing of the concept on cadaveric specimens, no reduction of the 3D model derived from patient CT data will be performed.

#### Positioning of the template and implant

**Fig. 2:**
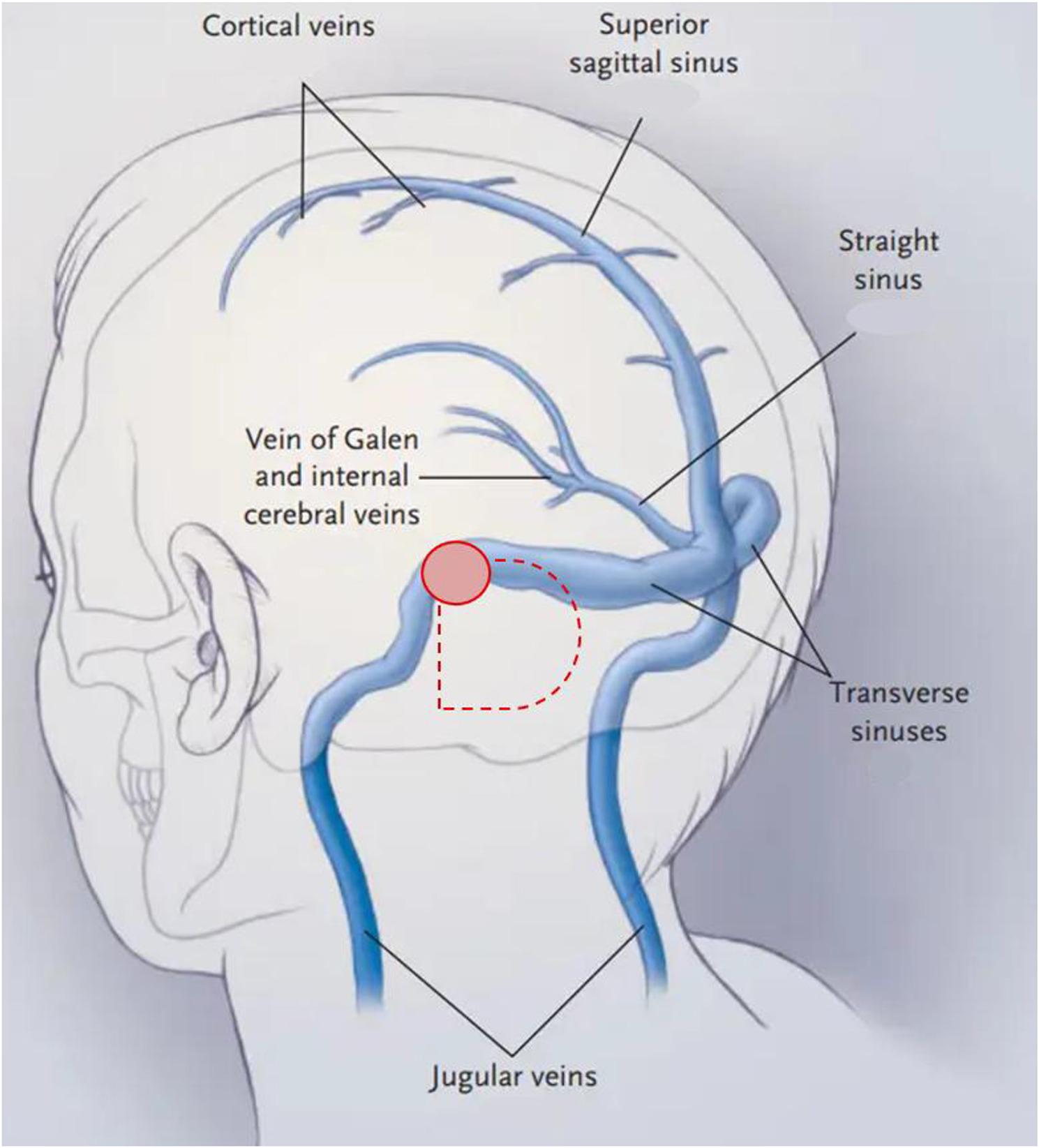
Schematic illustration of the positioning of the template and implant

The most critical aspect is the location of the surgical access point, and consequently, the positioning of both the template and the implant. Planning should be performed based on CT and MRI data, using neuronavigation, and in close collaboration with the surgeon. The burr hole is positioned directly over the sinus angle. The craniotomy is performed in a “D”-shaped configuration, with a vertical cut along the mastoid. The size of the planned template and implant is determined by the maximum extent of the surgical field, which in this study was 4 × 4 cm. Figure 2, modified from Stam 2005, schematically illustrates the location of the template and implant, including the positioning of the burr hole (red circle).

**Fig. 3:**
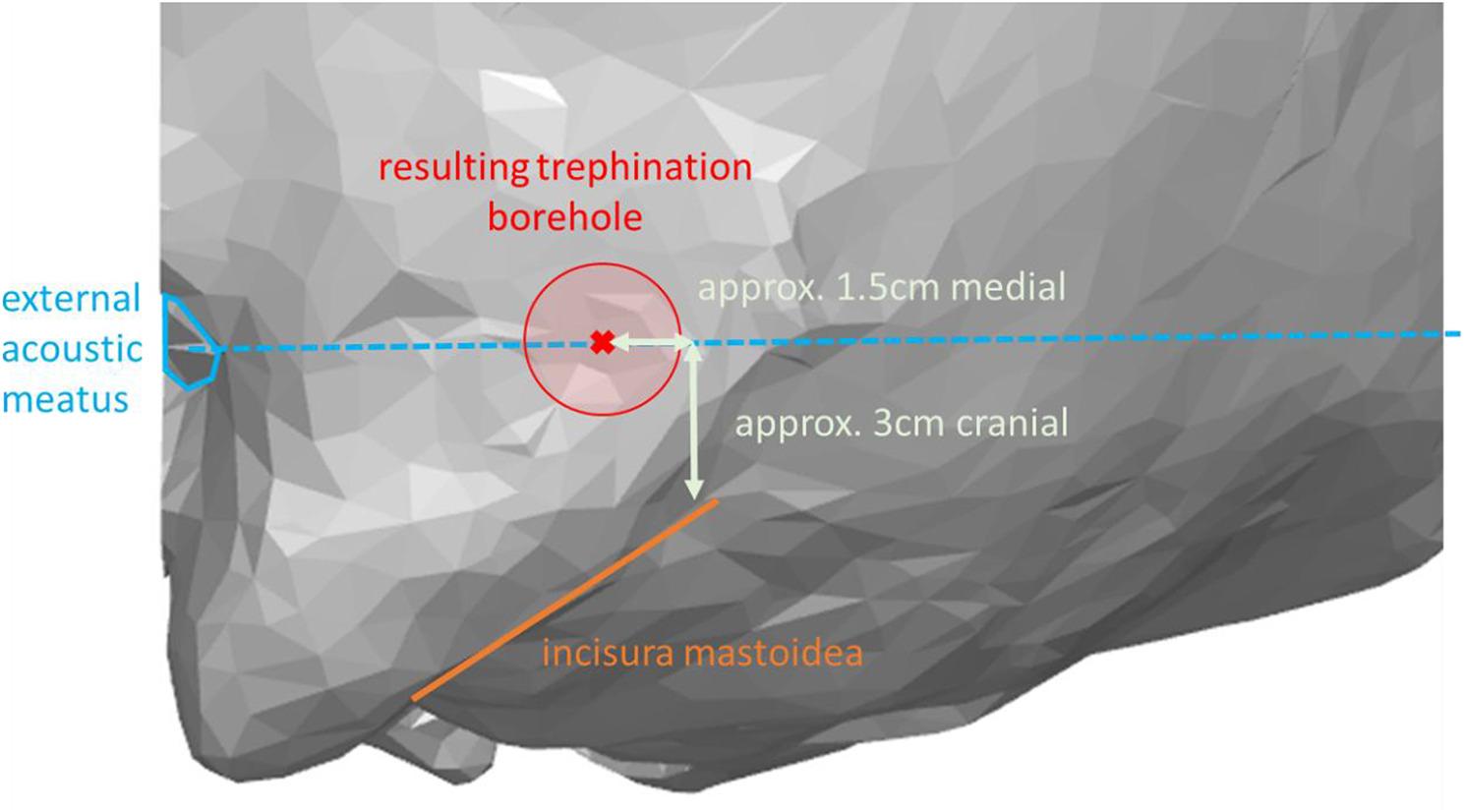
Positioning of the trephination hole

For the present study, neither MRI data nor neuronavigation were available. Therefore, planning was based on externally visible anatomical landmarks and the internal contours of the petrous bone. The center of the burr hole was positioned at the level of the external auditory canal, 3 cm cranial and 1.5 cm medial to the incisura mastoidea (Fig. 3).

Care was taken not to damage the petrous bone. The positioning was performed in consultation with a physician.

#### Template design requirements

The template must be user-friendly and serve as a universal solution for the entire craniotomy, meaning that various tools (e.g., craniotome, bone saw, and drill) can be used without requiring different templates. The positioning of the template should be straightforward. It is intended to be placed on the skull as per the surgeon’s guidance, with its contours traced onto the bone. Initial designs do not require the template to be fixed to the skull.

In such surgical procedures, it is often necessary to enlarge the opening to enhance visualization of anatomical structures or to facilitate more direct access. The template must therefore allow for minor extensions of the opening, while ensuring that both the template and the implant are designed to maintain complete coverage of the defect, even after enlargement.

#### Implant design requirements

In the design of the implant, it was imperative to ensure complete coverage of the defect, in addition to sufficient thickness to provide stability. The thickness of the skull in this area varies significantly, ranging from a few millimeters to up to 2 cm. Following extensive consultations with the neurosurgeon, an implant thickness of 5 to 8 mm was determined, aligning with the thickness of other comparable implants.

### Development of the template-implant concept

**Fig. 4:**
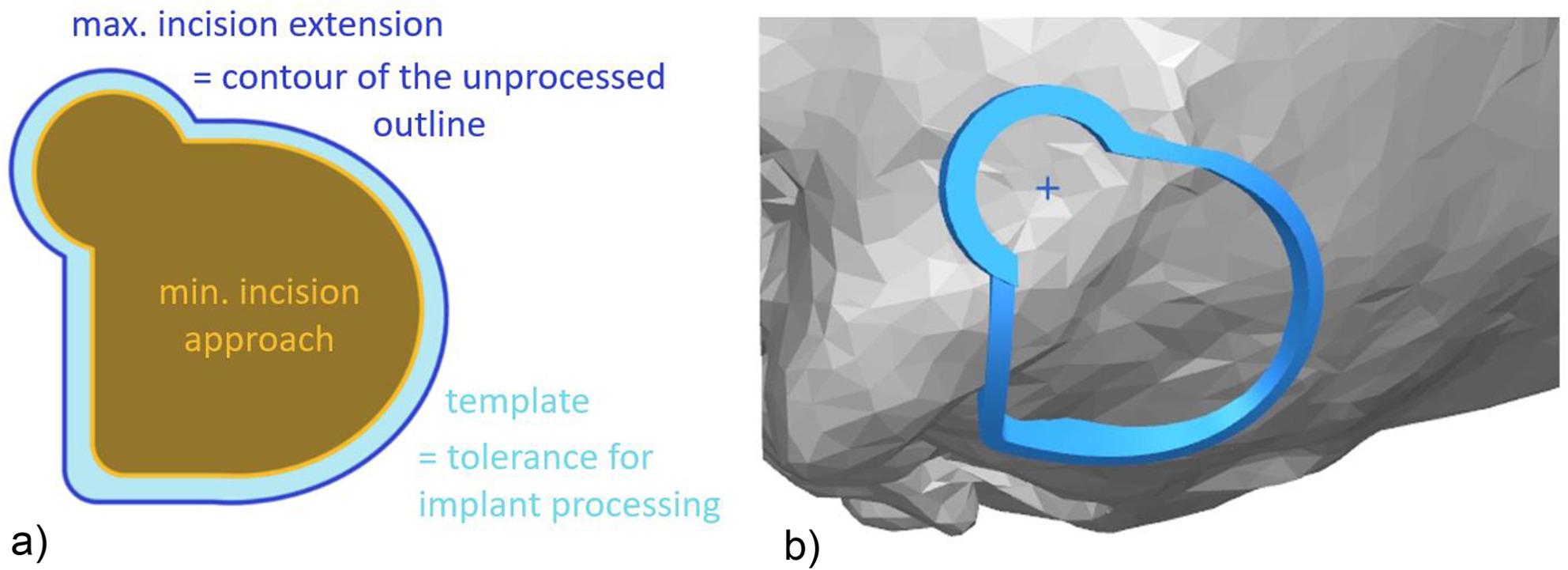
Concept of the template and implant (**a**) and positioning of the template on the skull (**b**)

The template and implant were designed based on the defined requirements. The surgical guide has been meticulously crafted to ensure that its internal surface conforms to the contours of the skull, facilitating precise positioning through straightforward placement on the bone. It is imperative to note that accurate positioning is only ensured when the guide is fully seated on the cranial surface. This feature enables the surgeon to verify the reproducibility of the implant’s position. The template is designed as a linear surface in the form of a frame. The inner edge (Fig. 4(a) - yellow line) of the template delineates the area to be excised during the craniotomy, which should be completely removed. Undercutting of this contour by the surgeon was excluded based on the space required during the procedure. The outer edge of the template (Fig. 4(a) - dark blue line) delineates the maximum possible extension, which should not be exceeded. The template’s configuration is a “D” shape, as specified in the stipulated requirements. The developed template is illustrated in Fig. 4(b). 

The implant itself was developed based on the designed template, with the outer contour of the template (Fig. 4(a) – dark blue line) corresponding to the outer contour of the implant. Unlike the template, the implant was fully enclosed to allow complete coverage of the defect, and thus, the size of the implant corresponds to the maximum extent of the craniotomy. If the craniotomy is smaller than the outer contour of the template, the implant can be adjusted to match the cutting lines. For this purpose, an auxiliary guide was developed to transfer the inner contour of the template onto the implant. This line must not be undercut during post-processing, as it represents the minimum size of the craniotomy. Undercutting this line during post-processing would prevent complete coverage of the defect.

**Fig. 5:**
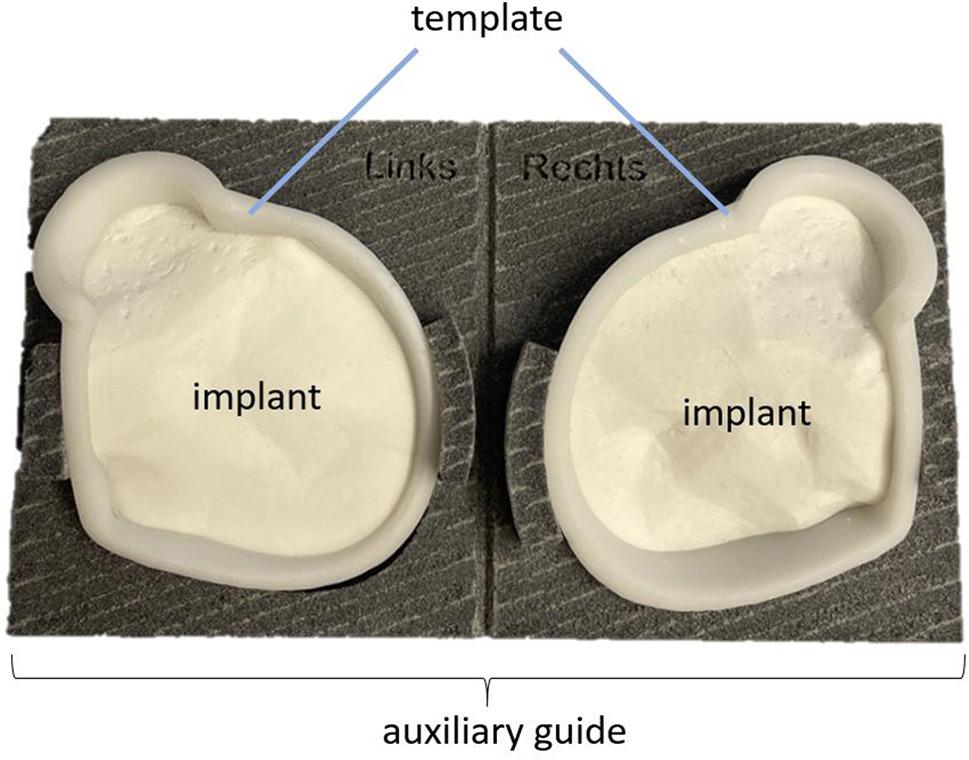
Auxiliary guide including template and implant

The implant is initially placed into the guide, followed by the positioning of the template on top (see Fig. 5).

### Materials and manufacturing processes

In clinical applications, it is imperative that the materials utilized are biocompatible. For the implant, an established material such as PEEK (polyetheretherketone) was considered; however, due to cost considerations and the employment of a simulation model, PEEK was not used in this preclinical feasibility study and must be evaluated in future studies. Instead, *PLA* (polylactic acid) from *Polymaker (Changshu*,* China)* was selected as the material. The fabrication of the implants was carried out through material extrusion, specifically the FDM (Fused Deposition Modeling) process, employing an *UltiMaker S7* 3D printer from *UltiMaker (Utrecht*,* Netherlands)*. Due to the layer-by-layer construction process and the necessity for support structures, the implants required post-processing to attain a smooth surface. This post-processing was executed through the utilization of small milling tools and sandpaper.

The templates were manufactured using a biocompatible resin, *Biomed White* from *Formlabs (Somerville*,* USA)*. The manufacturing process involved inverted vat polymerization, specifically the stereolithography (SLA) process, and was carried out using a Formlabs 3BL printer from *Formlabs (Somerville*,* USA)*.

For the auxiliary guide used to transfer the inner contour of the template to the implant, the powder bed fusion process was applied. The material utilized for this purpose was PA 12 (polyamide 12) from *HP (Palo Alto*,* USA)*, and the printer employed for this process was the *MJF 5200* also by *HP*.

#### 3D printed simulation model as a test object

**Fig. 6:**
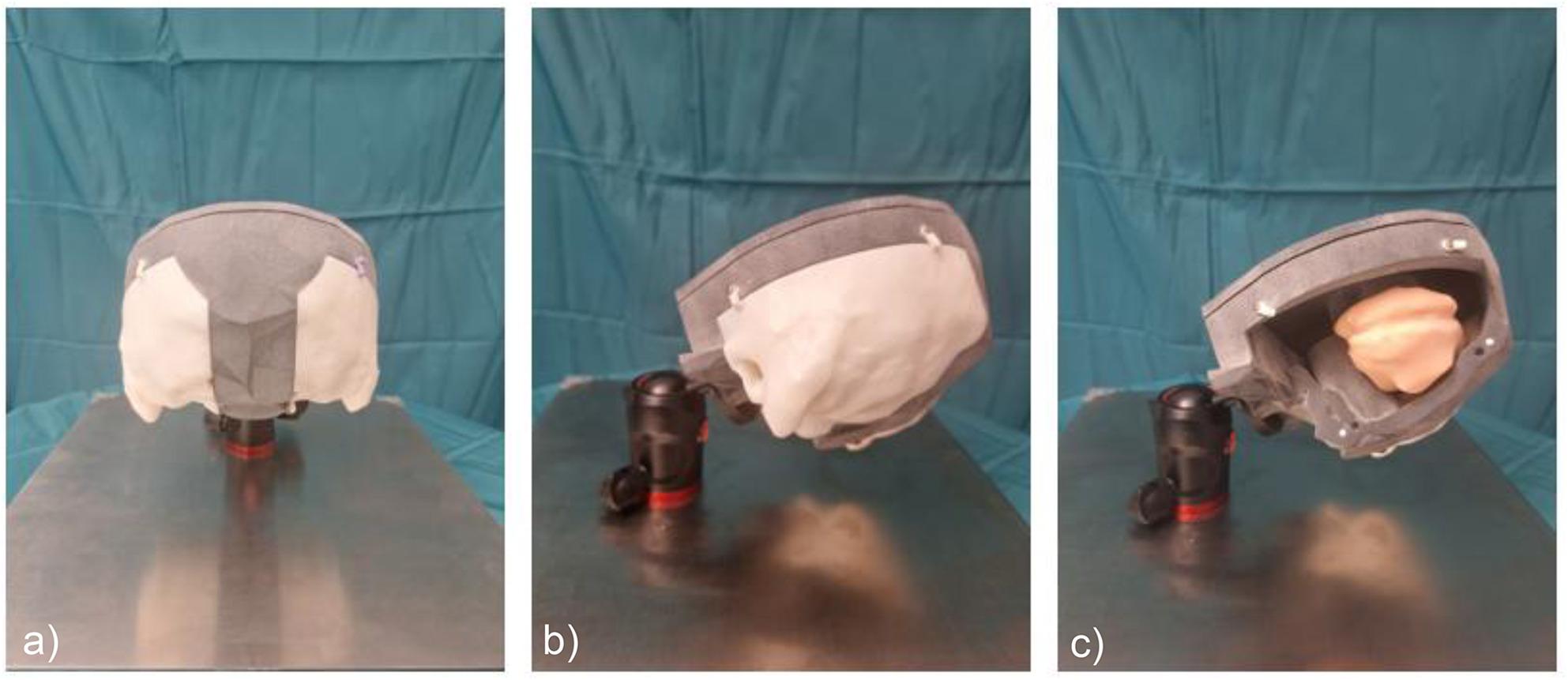
Representation of the 3D printed phantom. **a**) Posterior view. **b**) Lateral view. **c**) Lateral view with a perspective of the cerebellum

The novel concept was tested using a 3D printed phantom (Fig. 6), which was designed to replicate the posterior cranial fossa on both sides of the skull. The phantom incorporated interchangeable modules, enabling its application to all tests, with only the modules being replaced for each participant. Additionally, a cerebellum was integrated into the phantom to provide orientation and define the surgical area for subsequent operations. The fabrication of the phantom involved the integration of four distinct materials, three 3D printing techniques, and one casting process. The components, along with their respective materials and manufacturing methods, are enumerated in Table 1.

**Table 1:** Presentation of the manufacturing method and the materials used for each component of the phantom

Manufacturing method	Component/ object	Printer	Material
Inverted vat polymerization (SLA)	Interchangeable modules (both sides)	Form 3BL (Formlabs - Somerville, USA)	White V4 (Formlabs - Somerville, USA)
Material extrusion (FDM)	Clamping brackets	UltiMaker S7 (UltiMaker - Utrecht, Niederlande)	PLA (Polymaker - Changshu, China)
Powder bed fusion (MJF)	Housing	MJF 5200 (HP - Paolo Alto, USA)	PA 12 (HP - Paolo Alto, USA)
	Mounting of the cerebellum		
	Mold for the cerebellum		
Silicone casting	Cerebellum	-	Dragon Skin^TM^ 30, Slacker™, Ease Release^TM^ 200 (Smooth-On - Macungie, USA)

To ensure sufficient stability during testing, the phantom was mounted on a metal plate using a *Manfrotto MH494* camera tripod *(Cassola*,* Italy)*. The camera tripod provided the phantom with adequate support and high mobility, which was essential for positioning during the tests.

### Evaluation of printing accuracy

In order to conduct a precise evaluation of the novel concept, a comparative analysis between 3D-printed objects and virtual models was performed to assess printing accuracy. This encompassed the templates, implants and the interchangeable modules employed for testing purposes. The phantom housing was not included in the analysis, as its primary function was to serve as a holder for the interchangeable modules. It neither came into direct contact with the novel concept nor underwent modification during testing. The 3D-printed objects were then digitized using a Keyence scanner (VL-700). The GOM Inspect 2019 software was utilized to overlay and compare the physical models with the CAD data. Red areas indicate material excess relative to the CAD model (positive deviation), while blue areas indicate material deficit.

**Fig. 7:**
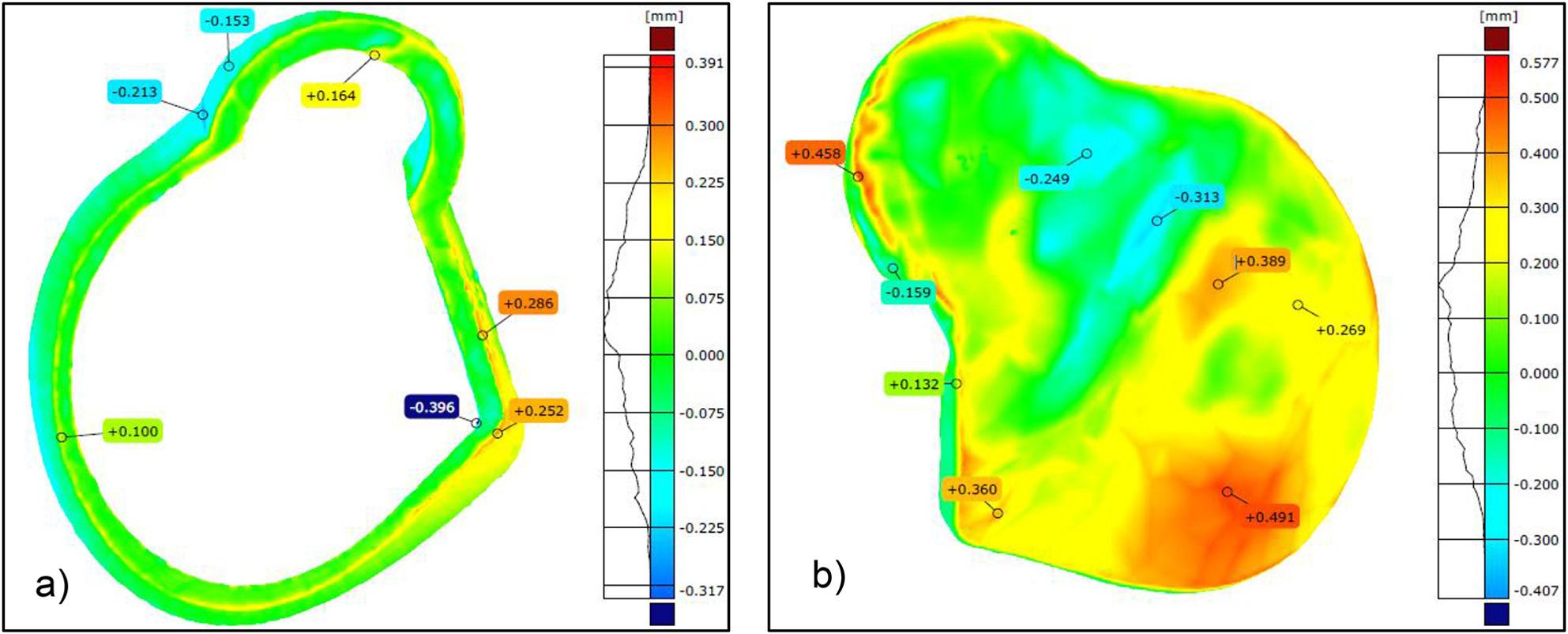
Comparison of the virtual and physical models. (**a**) Template. (**b**) Implant

Figure 7a illustrates the template, oriented with the lower edge visible, which later rests on the skull during marking. It was manufactured using inverted vat polymerization (stereolithography) and positioned to avoid support structures on the skull-contacting surface, ensuring a smooth finish. The implant in Fig. 7b was fabricated through material extrusion, specifically FDM. The depicted outer surface conforms to the skull anatomy. The surface facing the observer was oriented toward the print bed, placing support structures externally and preserving a smooth internal surface for brain contact.

**Fig. 8:**
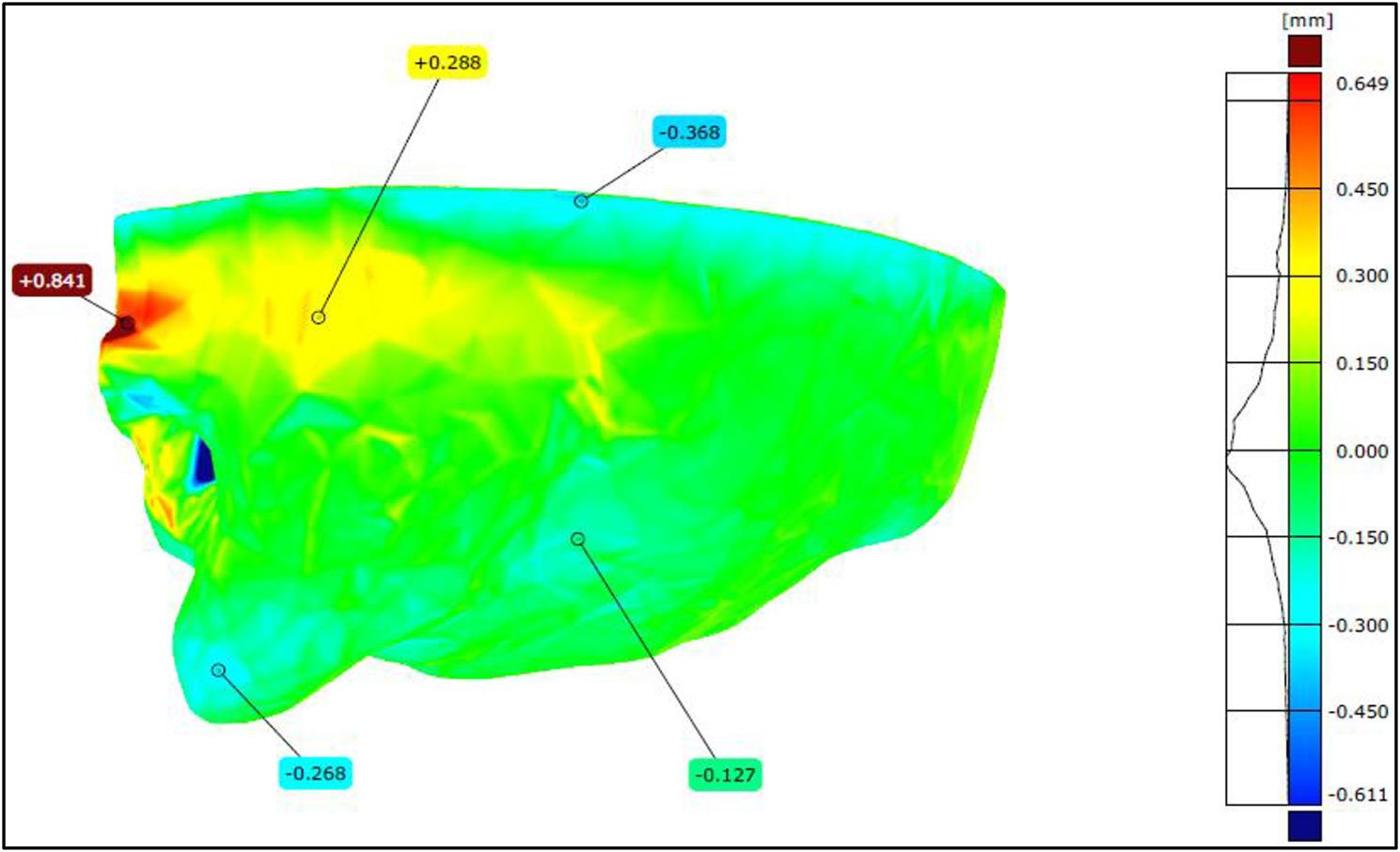
Comparison of the virtual and physical model of the interchangeable module

Figure 8 depicts an interchangeable module with the outer contour facing the observer.

This interchangeable module was also produced via stereolithography and oriented with its long edge (shown at the top of the image) toward the build platform, where support structures were attached.

The analysis indicates that discrepancies between printed and virtual models are negligible, with measurements consistently falling below one millimeter. These discrepancies may result from several factors. First, digitization using a Keyence scanner required merging multiple scans, which may introduce apparent offsets not present in reality. Second, post-processing steps such as support removal and surface smoothing can slightly reduce material. Additionally, stereolithography parts may retain minor resin residues after washing and curing, leading to small deviations. These minor deviations are not expected to significantly affect the results.

### Current standard of care based on patient data

**Fig. 9:**
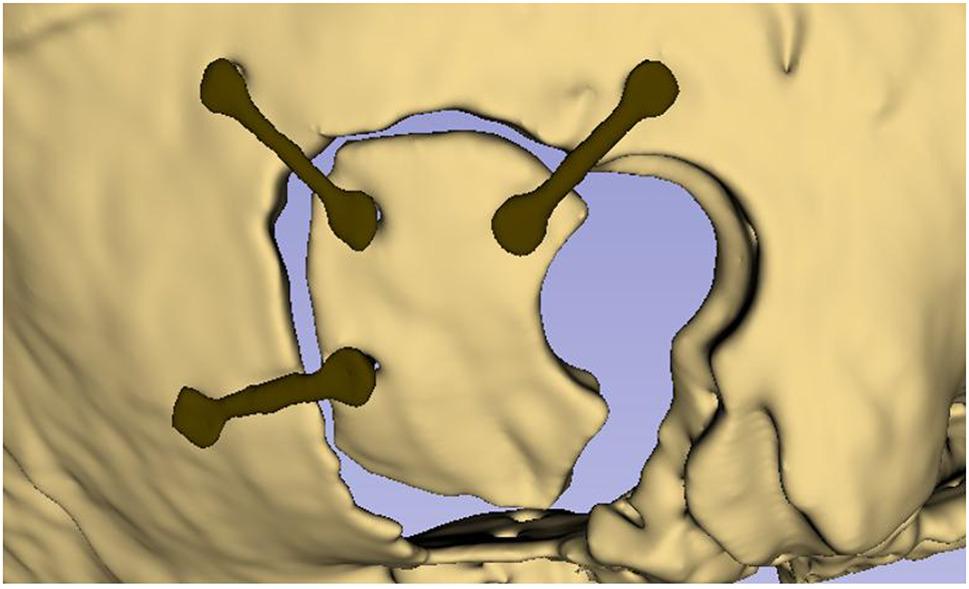
Illustration of the contemporary treatment modality for a craniotomy in the area of the posterior fossa

The novel concept was compared with the current standard of care (reimplantation of the removed bone fragment) using postoperative CT scans (slice thickness: 1–1.25 mm) from patients following the resection of vestibular schwannomas. The CT data were segmented using a Hounsfield unit threshold ranged between 220 and 3000 and analyzed to record the gap measurements between the edges of the skull and the reinserted bone, allowing for an evaluation of how well the bone covered the defect. A gap measurement of 0 mm would indicate complete coverage of the defect. A total of 329 measurements were recorded from 58 patients between 2012 and 2021. Figure 9 provides an example of the current method of covering a defect.

The determination of gap measurements was conducted by acquiring a minimum of four and a maximum of seven measurement points per patient. Due to the absence of standardization in the approach and the individualized extension of the craniotomy, it was not feasible to obtain a uniform number of measurements. To ensure an accurate assessment of the average gap, the largest (burr hole) and smallest gap measurements were recorded, along with at least two additional measurements. The additional measurements were selected to cover the entire perimeter of the reimplanted bone. In cases where the bone fragment was tilted, resulting in edges protruding above the skull surface, additional gap measurements were taken in these areas. This was not necessary for every specimen, which resulted in a varying number of measurements.

### Test setup and procedure for testing the novel concept

A test series was conducted on the 3D printed phantom to determine the gap measurements for the new concept, with the objective of comparing the concept of the template and the corresponding implant with the current standard of care. Nine specialists and senior physicians from the Department of Neurosurgery at the University Hospital Leipzig were asked, to test the novel concept on the phantom. For this purpose, the participants were provided with the novel template-implant concept, the simulation model, and a pen for marking, as well as a craniotomy set from *Aesculap AG* (*Tuttlingen*,* Germany*). The trephine attachment used was a *Disposable Cranial Perforator with Hudson End (11/14 mm | 3 mm)* from *EMD-MED* (*Debrecen*,* Hungary*). Additionally, they were provided with a *CRS-M spiral drill* from *adeor medical AG* (*Valley*,* Germany*) as the cutting tool for the bone drill.

Prior to testing, the participants received a brief explanation of the new concept. They were then asked to independently position the template and transfer its contours onto the phantom. After marking the inner and outer radii of the template, it was set aside. The craniotomy was subsequently performed using the trephine and bone drill. The marked radii provided orientation for the boundaries within which the craniotomy should be performed. After completing the craniotomy, the previously fabricated implant was inserted. The implant had to be adjusted to match the performed craniotomy. The participants were assisted by the template and an additional drill to refine the edges of the implant. Once prepared, the implant was fixed to the phantom using plates and screws. The time required for template placement, craniotomy, and post-processing and insertion of the implant was recorded to estimate the additional procedural effort associated with the novel concept during surgery.

**Fig. 10:**
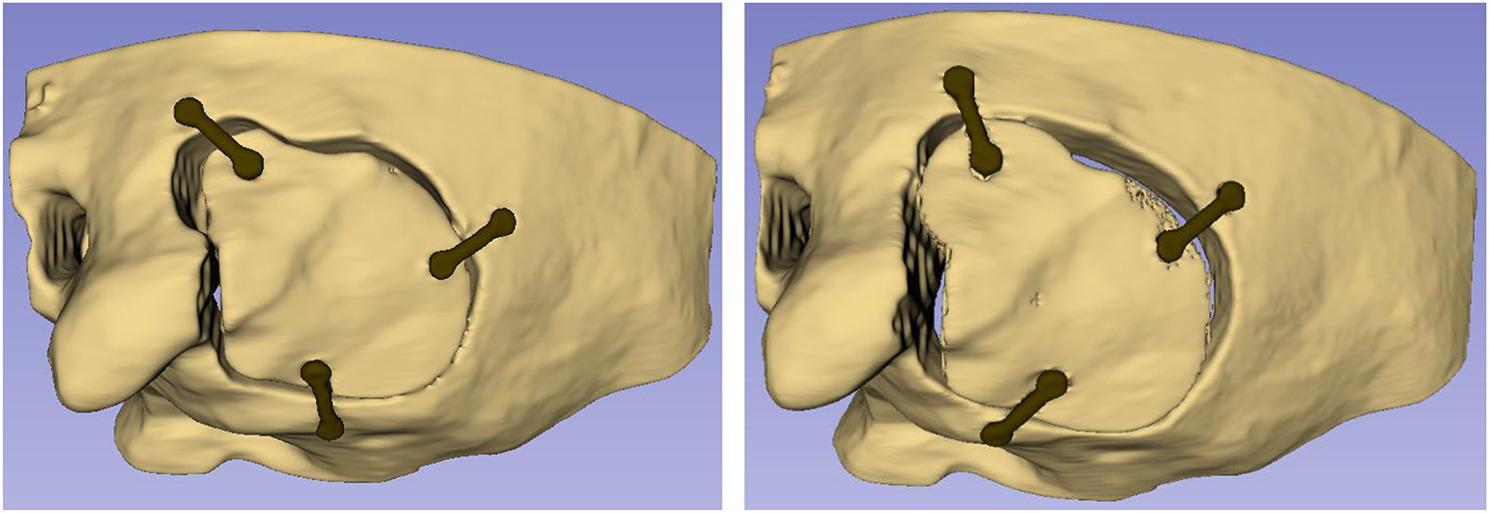
Representation of two models generated from CT scans (left – Participant 2, right – Participant 8)

**Fig. 11:**
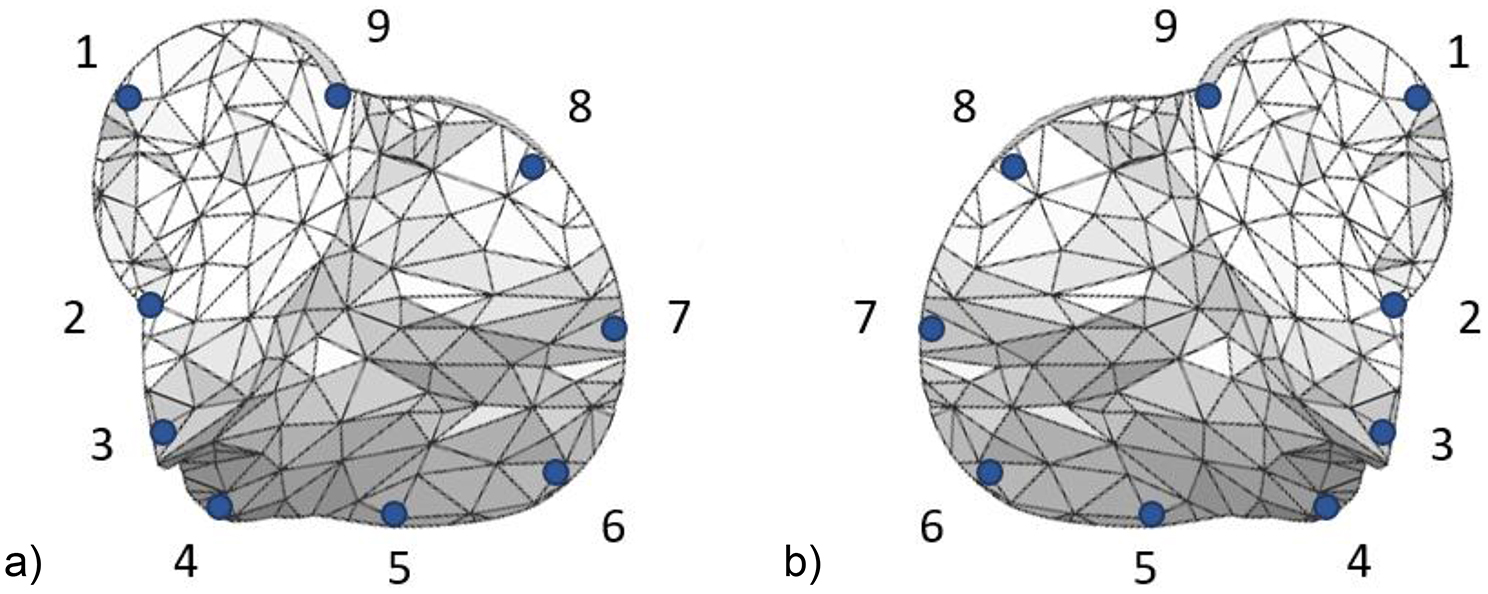
Definition of the measuring points on the implant fort he left side (**a**) and the right side (**b**)

Each neurosurgeon performed the craniotomy on both sides of the phantom, resulting in a total of 18 craniotomies. The evaluation phase involved the acquisition of CT scans (slice thickness: 0.625 mm) of the interchangeable modules within the phantom, including the implanted devices (Fig. 10). These data were subsequently loaded into the open-source software *3D Slicer (Boston*,* USA)*, and the gap measurements were obtained. Nine points on the implant that were clearly identifiable for this purpose were defined, and these points are shown in Fig. 11.

## Results

In order to evaluate the data from the current standard of care, the average gap measurement was calculated from the 329 recorded values. The median and standard deviation were also determined (see Table 3).

For the novel concept, a similar approach was applied. Utilizing the nine delineated measurement points and two craniotomies per participant, a potential of 162 gap measurements could have been generated. However, one participant was only able to complete the procedure on one side due to an incomplete craniotomy that prevented the removal of the “bone flap.” Consequently, 154 gap measurements were collected (see Table 2).

**Table 2:** Measurements of the gap dimensions for each participant on both sides

	**Gap dimensions at the measuring points [mm]**										
			**P 1**	**P 2**	**P 3**	**P 4**	**P 5**	**P 6**	**P 7**	**P 8**	**P 9**
**Participant**											
	1	left	0,84	3,45	3,53	1,08	2,58	1,56	0,64	0,80	1,52
		right	1,36	1,47	0,10	9,17	4,57	1,64	0,19	0,65	2,37
	2	left	2,66	1,48	2,61	2,32	1,82	0,84	0,96	2,06	1,36
		right	1,57	3,52	2,66	0,91	4,09	1,03	0,77	1,49	0,79
	3	left	3,22	1,85	0,86	1,81	1,89	0,96	2,03	1,12	3,36
		right	0,57	3,82	3,12	2,15	3,21	1,00	0,97	1,82	2,35
	4	left	1,58	4,83	1,52	1,29	2,17	0,76	2,93	0,73	2,78
		right	3,00	1,54	3,33	6,32	5,80	4,45	0,55	0,98	4,43
	5	left	1,79	4,02	1,45	3,05	3,07	0,97	0,25	1,56	2,83
		right	1,65	4,52	3,56	1,89	3,78	1,75	1,64	1,63	1,25
	6	left	1,20	6,72	3,39	2,77	2,95	0,75	0,71	0,90	2,63
		right	N/A								
	7	left	5,16	2,54	2,95	1,48	1,56	0,50	1,80	2,80	6,37
		right	9,87	4,52	2,93	1,90	3,52	2,06	0,33	2,34	3,38
	8	left	2,27	4,36	3,80	2,09	1,52	0,77	1,92	3,74	4,48
		right	0,72	2,63	2,91	0,07	0,74	1,70	1,56	2,44	2,89
	9	left	2,86	4,14	4,27	1,30	2,65	1,74	0,80	1,38	4,01
		right	3,17	4,61	2,98	1,23	2,83	0,97	1,42	1,70	3,42

For each participant and side, the average of the gap measurements was calculated. The median and standard deviation across all recorded values were subsequently determined. The calculated values for both the current standard and the novel concept are summarized in Table 3.

**Table 3:** Comparison of the current supply and the new concept

	Gap dimensions			
	number of measurements	median [mm]	average [mm]	standard deviation [mm]
current standard	329	5.52	6.17	3.80
new concept	154	2.11	2.46	1.54

**Fig. 12:**
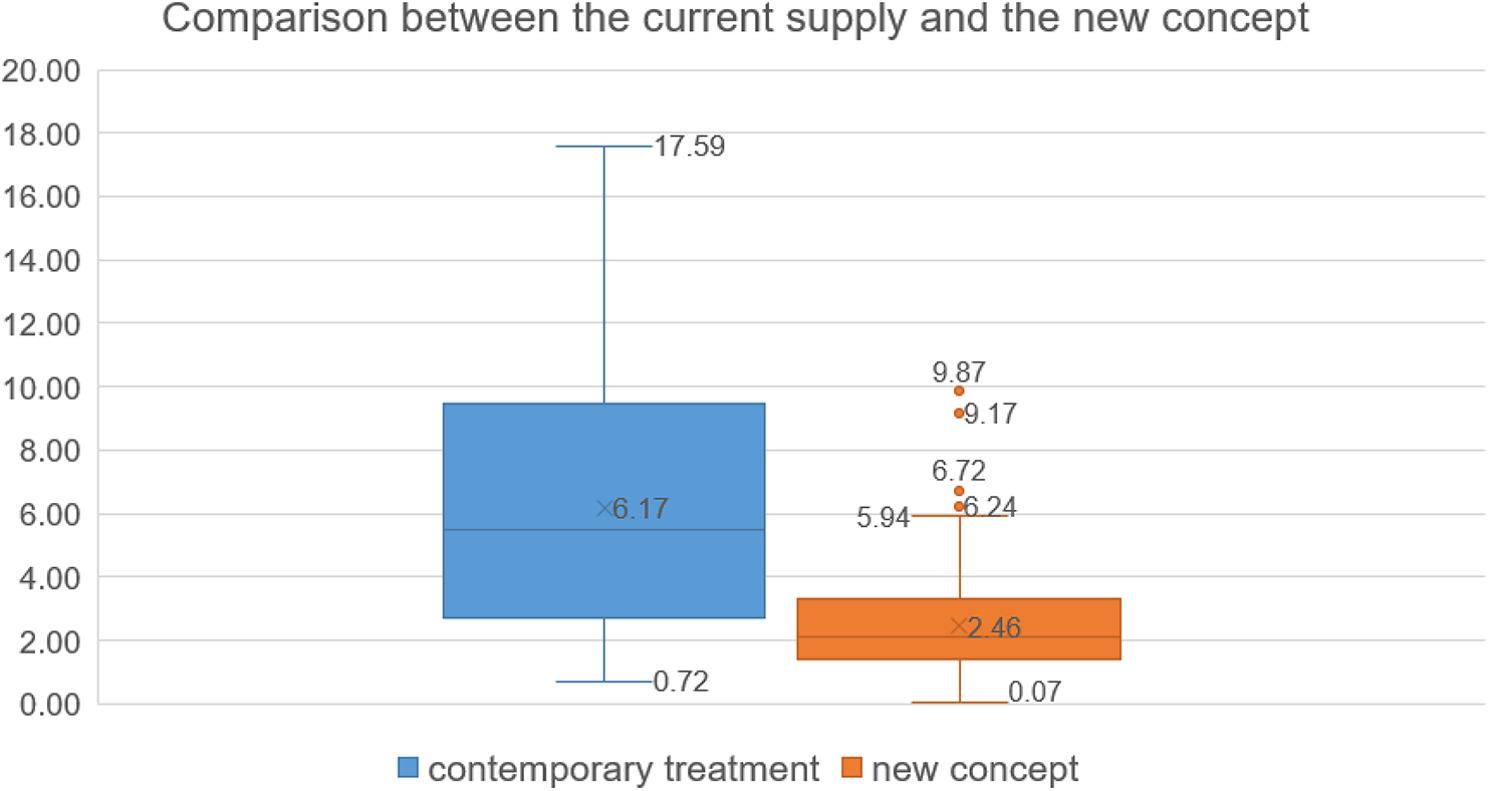
Comparison between the contemporary treatment (blue) and the newly developed concept (orange)

To facilitate the interpretation of the results, the generated data from the current standard of care and the novel concept were represented in a boxplot diagram (see Fig. 12).

The calculated values and graphical representation demonstrate that the values of the novel concept exhibit reduced variability compared to the current standard of care (width of the box). Furthermore, the average gap measurements when using a template with a matching implant are significantly smaller (> 3.5 mm). It is also apparent that the maximum measured gap sizes differ significantly (current standard of care: 17.59 mm, new concept: 9.87 mm). In a similar vein, the minimum values exhibit variability. While both whiskers are situated below 1 mm, the new concept demonstrated superiority with a minimum gap width of 0.07 mm compared to the prevailing standard of care, which is 0.72 mm.

In addition to the gap measurements, the times required for preparation—i.e., for positioning the template and craniotomy—as well as for post-processing and implant placement were recorded. Table 4 presents the individual times per participant and side, as well as the resulting additional time that would be required during surgery.

**Table 4:** Listing of individual times for each participant per side, as well as the overall average, for preparation and craniotomy, and for post-processing and implant placement

				Time required using the template-implant concept [min]	
				Preparation and craniotomy	Post-processing and implant placement
**Participant**		1	left	5	12
			right	6	8
		2	left	4	6
			right	4	6
		3	left	8	16
			right	11	7
		4	left	4	15
			right	5	8
		5	left	6	3
			right	4	5
		6	left	8	5
			right	N/A	
		7	left	6	6
			right	4	3
		8	left	8	12
			right	3	4
		9	left	7	8
			right	5	5
				Ø = 5.76	Ø = 7.59

The table shows that preparation, including positioning and transferring the template, as well as opening of the skull, took on average approximately 5:45 min. Post-processing and implant placement were slightly more time-consuming, averaging around 7:35 min. Summing the times for preparation and post-processing provides the additional time required for the new concept, including the duration of the craniotomy and fixation of the implant to the skull. The average total time was 13:20 min.

## Discussion

The present results demonstrate that the developed template-implant concept enables a significant reduction of gap sizes compared to the current standard of care. This improvement can be explained by the fact that in the conventional approach, the resected bone fragment is reimplanted, which, due to the craniotomy diameter and potential intraoperative extensions, is always smaller than the craniotomy itself. Consequently, a gap of approximately 15 mm (the diameter of the trephination borehole) remains, which can be fully covered by the new implant concept. In addition to reducing gap sizes, preoperative planning and the individual design of the template help prevent incomplete defect coverage caused by intraoperative extensions. The lower variability observed in the results is likely due to testing being conducted on a single phantom using identical templates and implants. By contrast, the comparison dataset comprised CT scans from 58 different patients, representing craniotomies of varying sizes and positions, which naturally increases variability. Therefore, a clinically meaningful assessment of the concept can only be obtained through studies involving multiple cadaver specimens.

**Fig. 13:**
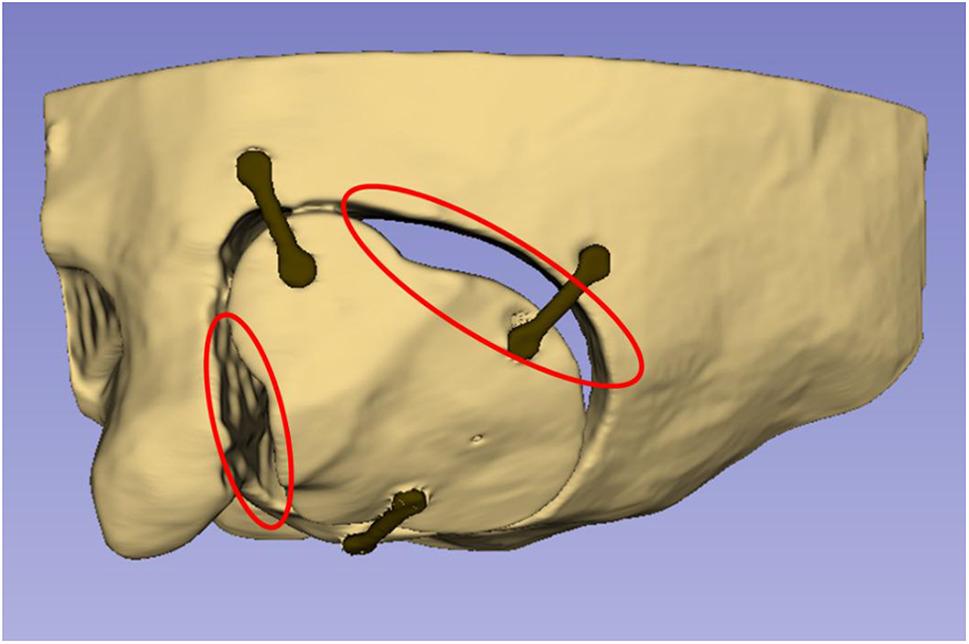
Edited model (left) of participant 8 showing the resulting defects caused by an altered cutting contour and/or modification of the skull instead of the implant (red circles).

At the same time, the results also highlight limitations of the approach. Despite identical initial conditions, differences between participants were observed. These differences are primarily attributable to the handling of the template, which served only as a marking guide during the craniotomy and was not fixed as a cutting edge. The exact decision regarding the cut was left to the surgeon, which led to extensions that could no longer be covered by the implant. A handling issue was also observed, as in many cases the skull rather than the implant was worked on. This was likely because the fixed skull offered greater stability than the small, hand-held implant, but it led to unintended additional defects (e.g., Fig. 13 – red circles).

Another critical aspect is the positioning of the template. In the present preclinical feasibility study, only reduced CT datasets without accompanying MRI data were available, preventing orientation relative to internal structures such as the sinus angle. As a result, placement relied solely on externally visible landmarks, increasing positional uncertainty. For clinical translation, the combined use of CT and MRI data is essential, and planning should be conducted in close collaboration with the surgeon. This is particularly important because, unlike the reduced phantom model, a real human skull has a considerably smoother surface, and mastoid prominence varies among patients, making precise template placement more challenging in cases with less pronounced mastoids or flatter skulls. In clinical practice, the retrosigmoid approach is routinely planned and guided by neuronavigation, which can support accurate template positioning and reduce intraoperative variability. However, further cadaveric studies are needed to validate and optimize the reproducibility and clinical applicability of the approach.

Table 4 shows that the preparation and post-processing of the procedure, including opening and closing the skull, took less than 15 min on average. Assuming approximately 5 min each for the craniotomy and reinsertion of the bone flap or implant placement, the template-implant concept required an additional procedural effort of around 5 min. Complete defect closure using the current standard method, which involves reimplantation of the resected bone, often requires bone cement that can be molded directly onto the defect. Compared to the new concept, the use of bone cement is more time-consuming due to mixing, handling, and curing, taking approximately 15 min [27]. A comprehensive assessment of the advantages of the template-implant concept over defect coverage with bone cement therefore requires further clinical studies.

The current standard method at the University Hospital Leipzig involves reimplantation of the bone flap without additional defect closure using bone cement. In comparison, the novel concept offers superior coverage of the defect. A disadvantage is the increased preoperative planning effort. Early acquisition of imaging data is required for the production of the template and implant, and the manufacturing process can take several days up to one week, depending on the PEEK printer used. Furthermore, the costs of currently € 6,000–9,000 per patient-specific PEEK implant, including the template, represent a potential limitation for clinical implementation.

## Conclusion

The newly developed concept combining a template and a patient-specific implant demonstrates significant potential to improve defect coverage in retrosigmoid approaches by achieving smaller gap sizes. Preoperative planning and the individual adjustability of the template contribute to minimizing intraoperative extensions and ensuring complete defect coverage. At the same time, the concept illustrates how patient-specific implants produced using 3D printing technologies can enhance surgical precision and procedural predictability.

Despite these advantages, relevant limitations exist, particularly regarding intraoperative handling, precise positioning without complete imaging data, preoperative time requirements, and the costs associated with patient-specific PEEK implants. Future investigations using cadaveric specimens and clinical studies are necessary to validate reproducibility, efficiency, and clinical safety. Through precise planning and individualized adaptation, the template-implant system optimizes the surgical workflow, improves patient outcomes, and demonstrates the potential of additive manufacturing technologies in medicine.

## Data Availability

The data are given in the paper and can be received on request.
